# User Association and Power Control for Energy Efficiency Maximization in M2M-Enabled Uplink Heterogeneous Networks with NOMA

**DOI:** 10.3390/s19235307

**Published:** 2019-12-02

**Authors:** Shuang Zhang, Guixia Kang

**Affiliations:** 1 Key Laboratory of Universal Wireless Communications, Ministry of Education, Beijing University of Posts and Telecommunications, Beijing 100876, China; zhangshuang2015@bupt.edu.cn; 2 Wuxi BUPT Sensory Technology and Industry Institute CO. LTD, Wuxi 214000, China

**Keywords:** M2M, heterogeneous networks, non-orthogonal multiple access, energy efficiency, MU association, power control

## Abstract

To support a vast number of devices with less energy consumption, we propose a new user association and power control scheme for machine to machine enabled heterogeneous networks with non-orthogonal multiple access (NOMA), where a mobile user (MU) acting as a machine-type communication gateway can decode and forward both the information of machine-type communication devices and its own data to the base station (BS) directly. MU association and power control are jointly considered in the formulated as optimization problem for energy efficiency (EE) maximization under the constraints of minimum data rate requirements of MUs. A many-to-one MU association matching algorithm is firstly proposed based on the theory of matching game. By taking swap matching operations among MUs, BSs, and sub-channels, the original problem can be solved by dealing with the EE maximization for each sub-channel. Then, two power control algorithms are proposed, where the tools of sequential optimization, fractional programming, and exhaustive search have been employed. Simulation results are provided to demonstrate the optimality properties of our algorithms under different parameter settings.

## 1. Introduction

The increase of smartphones, laptops, and other mobile devices as well as data-hungry applications, need huge demands for ubiquitous coverage and very high data rates in cellular networks. However, homogeneous networks cannot satisfy these requirements [1]. Then, two-fold efforts have been spent to meet the stringent requirements. On one hand, researchers have proposed heterogeneous networks (HetNets) where different types of base stations (BSs), e.g., macro BSs (MBSs) and small BSs (SBSs) are deployed in a multi-tier hierarchical structure. In this structure, all BSs have seamless coverage and reuse frequencies to achieve higher data rate [2,3]. On the other hand, the so-called non-orthogonal multiple access (NOMA) has been investigated as a potential technique to further improve the throughput of network [4,5,6,7]. Different from conventional orthogonal multiple access (OMA), NOMA serves multiple users at the same time/frequency/codes resource by allocating different powers for them, and the superposition coded signal can be decoded at receivers by successive interference cancellation (SIC). Therefore, the combination of HetNets and NOMA will exhibit great potential to satisfy the 1000-times increase of mobile broadband data for the upcoming fifth generation (5G) communication systems and beyond [3].

However, the severe inter-tier and intra-tier interference make the NOMA-enabled HetNets challenging to achieve. Resource management plays an important role to alleviate these interference [8]. For downlink communication, specifically, some work focuses on the sum rate maximization and shows higher spectral efficiency (SE) can be achieved by NOMA when considering the intercell interference [9,10,11,12]. Besides SE, energy efficiency (EE) is also a key performance metric investigated for resource allocation in NOMA-enabled HetNets [8,13,14]. Moreover, EE is more important in uplink than in downlink NOMA-enabled HetNets since the devices in uplink communications are often battery-limited. It is a fact that the battery capacity has been improved at a very slow pace over the past decades [15], and hence this increase cannot scale with the high energy consumption caused by the increasing traffic demands. Meanwhile, EE has emerged as a new prominent performance metric for wireless communication networks designs due to the economic, operational, and environmental concerns [16,17]. Therefore, it is a stringent work to improve EE for uplink transmission.

Machine-to-machine (M2M) communications, also known as machine-type communications (MTC), enable pervasive connections to support IoT. M2M communications are one of the potential applications of NOMA-enabled HetNets [18], since NOMA-enabled HetNets provide a practical infrastructure to offer massive access opportunities for such a huge number of devices, especially for the cases in which each device only needs to send a small amount of data periodically in uplink. One of the challenges for HetNets with M2M communications is the access control, which can manage the engagement of massive MTC devices (MTCDs) to the core network. Among the existing access solutions, deploying MTC gateways (MTCGs) is an effective approach to connect M2M communication and cellular communication [19,20,21]. When mobile user (MU) has more power and storage space than MTCDs (e.g., smart sensors), the MU can be configured as the MTCG, as proposed in [22].

Since 5G will be HetNets including various network models (e.g., cellular networks, wireless networks (WSNs), and low power wireless area networks) to support high data rate and massive devices [3], our work combing M2M communication and cellular network has a large significance for this heterogeneous scenario. The short distance communication in our system model can be realized with WSNs, which provide a new way to help the sink nodes in WSNs communicate to the core network. For example, the MTCDs can be the sensors in an environmental monitoring WSN, and they can transmit the collected data to the core network through a mobile device in cellular networks with NOMA. Therefore, our work also has a practical significance for sensors work.

Recently, there have been some studies addressing the aforementioned challenges of applying NOMA in HetNets for EE maximization. In [23], a distributed user association algorithm based on inter-cell interference plus noise ratios of BS and a centralized user association based on the popular size of BS were both proposed. After user association was determined, a power control algorithm was proposed based on Lagrangian dual method, then a one-dimensional search algorithm was used to search Lagrangian multiplier, which added algorithm complexity. Two specific examples were provided to demonstrate the effectiveness of unified NOMA-enabled heterogeneous ultra-dense networks with user association and power control in [18]. An alternated energy efficient resource allocation algorithm based on fixed power allocation was first proposed in [13]. Then, two iterative energy-efficient resource allocation algorithms were proposed to update for better EE based on Lagrangian dual method. Joint base station association and power control optimization algorithms were proposed based on coalition formation games and interior-point method in [24], but sub-channel allocation and fractional equation for EE maximization had not been considered. Moreover, the user association algorithms in the aforementioned work were all considered with fixed power allocation firstly, whereafter iterative algorithms were used to obtain the final optimal value.

There are also some studies on the usage of M2M communications in NOMA systems. For example, energy-efficient resource allocation with hybrid division multiple-access NOMA for cellular-enabled M2M communications was researched in [25,26]. With MTCDs cluster formation known beforehand, standard convex optimization and Lagrange duality methods were employed respectively for power control in [25,26]. User clustering in NOMA-aided cellular M2M communication systems was researched in [27,28] with millimeter-wave and narrow-band IoT separately. A joint power and sub-channel allocation for secrecy capacity algorithm was proposed in [29] to obtain the suboptimal solution of the optimization problem. However, the aforementioned work deploys M2M user in single-cell networks. The trend of more and more intensive network deployment motivates us to deploy M2M-enabled NOMA in the scenarios with multi-tier HetNets and new resource allocation needs to be considered with the non-convexity caused by inter-cell interference in HetNets.

In this paper, we focus on the uplink EE maximization via user association and power control for M2M-enabled HetNets using NOMA. In this scenario, one macro base station (MBS) is located in the cell center. Each small cell has one small base station (SBS) located in the cell center. MUs are distributed randomly in the cell. An MU acting as an MTCG can decode and forward both the information of MTCDs and its own data to the BS. The EE (bits/Joule) maximization problem is formulated and solved to obtain the optimal MU association and power allocation. The main contributions of this paper are summarized as follows:

- We propose a new framework of M2M-enabled HetNets with NOMA. In this framework, control data separation architecture, i.e., control information and data message are separated, which can reduce the signal overhead [30]. NOMA is adopted by the MTCDs to transmit the information to MUs which is regarded as the relay. MUs decode the overlaid information and simultaneously transmit received data to the BSs based on the NOMA principle.
- In order to solve the EE maximization optimization problem, a BS and a sub-channel are included in a couple, since a MU can only associate one BS at one sub-channel. Then, a many-to-one MU association algorithm is proposed based on matching game [31]. Through swap operation among each couple, the EE maximization problem can be tackled by solving the power control problem at each sub-channel. Compared with the previous studies on the algorithms (user association and power allocation) [13,18,23,24], our algorithms are jointly optimized and fixed power allocation is not required for initialization.
- Two power control algorithms are proposed based on sequential optimization [32,33]. The fractional programming [34] and sequential optimization are combined to develop a novel sequential fractional power control algorithm (SFPCA), from which the original problem is transformed to be convex and requires less computational complexity. The other algorithm combines the exhaustive search method with sequential optimization, which can verify the correctness of SFPCA.

The rest of this paper is organized as follows. The system model and problem formulation are focused in Section 2. The MU association matching algorithm is proposed in Section 3. The power control problem is solved in Section 4. Numerical results are provided in Section 5, and concluding remarks are given in Section 6.

Notations: Lowercase and uppercase boldface letters denote vectors and matrices, respectively. We use uppercase decorated letters to denote sets. For an arbitrary set M, we always have the corresponding uppercase *M* to the denote the cardinality of M, i.e., $\left|M\right|=M$, ${\left[\cdot \right]}^{T}$ denotes the transpose operator.

## 2. System Model and Problem Formation

### 2.1. System Model

**Figure 1:**
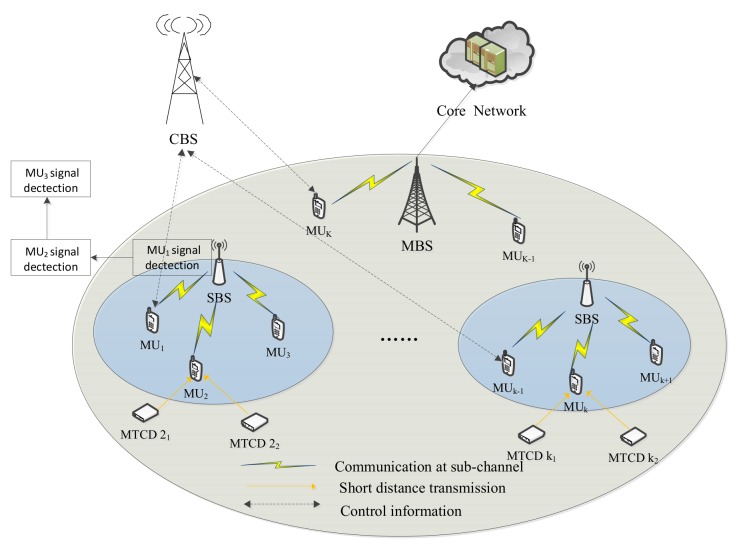
System Model.

As shown in Figure 1, we consider uplink HetNets with M2M communications, where all MUs are anchored to the control base station (CBS). The CBS performs the MU association algorithm to select the best serving BS for MUs and establishes a high BS–MU connection through backhaul links. Each MTCD selects the nearest MU as an MTCG. Since NOMA is adopted between MTCDs that select the same MU as their MTCGs, SIC is performed at the MU to gather the interference and channel gain will be obtained by channel estimation at the MU. The HetNets consist of a set $F=\left\{0,\cdots ,F\right\}$ of BSs and a set $K=\left\{1,\cdots ,K\right\}$ of MUs. Each MU is regarded as an MTCG, which can acts as a relay for some MTCDs. Denote $U_{k}$ as the specific set of MTCDs served by MU *k* (${\mathrm{MU}}_{k}$). The index 0 denotes the MBS and other indexes stand for the SBSs in set F. Without special explanation, we always have f∈F, $F=\left|F\right|$. The system bandwidth shared by all BSs is divided into *N* orthogonal sub-channels, and each one is assigned with bandwidth *B*. For convenience, hereinafter we always have n∈N={1,2,⋯N} to denote the sub-channel. MUs are served by BSs according to the BSs’ coverage.

### 2.2. NOMA Strategy

Multiple MTCDs can simultaneously transmit signals to the MU using NOMA. Since MUs and MTCDs use different transmission modes, we ignore the interference between MUs and MTCDs. The interference between MTCDs in different BSs is also not considered. According to the NOMA principle, the received signal of ${\mathrm{MU}}_{k}$ is

(1) $$Y_{k}=\sum_{j\in U_{k}}h_{j_{k}k}\sqrt{q_{j_{k}}}s_{j_{k}}+n_{k},$$

where $h_{jk}$ is the channel between MTCD $j_{k}$ and ${\mathrm{MU}}_{k}$; $q_{j_{k}}$ and $s_{j_{k}}$ denote the transmit power and message of MTCD $j_{k}$; and $n_{k}$ represents the additive zero-mean Gaussian noise with variance $\sigma^{2}$. $U_{k}$ represents the set of MTCDs which are served by ${\mathrm{MU}}_{k}$. Without loss of generality, the channels are sorted by h1kk2h1kk2σk2σk2>h2kk2h2kk2σk2σk2>⋯hUkk2hUkk2σk2σk2>0. Applying SIC in NOMA [33], the achievable data throughput for MTCD $j_{k}$ at ${\mathrm{MU}}_{k}$ is given by

(2) $$R_{j_{k}}=\log_{2}\left(1+\frac{H_{j_{k}k}q_{j_{k}}}{1+I_{j_{k}}}\right),$$

where Hjkk=hjkk2hjkk2σk2σk2, $I_{j_{k}}=\sum_{i\in \left\{U_{k}\left|H_{i_{k}k}\right.<H_{j_{k}k}\right\}}q_{i_{k}k}H_{i_{k}k}$, and we define $I_{j_{k}}=0$ for $j_{k}=U_{k}$. After MUs successfully decode the messages from MTCDs, all MUs simultaneously transmit data to the BS based on the NOMA principle. Denote $h_{kfn}=g_{kfn}\sqrt{d_{kf}^{-\alpha }}$ as the channel gain between ${\mathrm{MU}}_{k}$ and BS *f* at sub-channel *n* (${\mathrm{SC}}_{n}$). $g_{kfn}$ denotes the corresponding Rayleigh fading channel gain; α is the path loss factor; and $d_{kf}$ is the distance between ${\mathrm{MU}}_{k}$ and BS *f*. In order to split the superimposed signals on ${\mathrm{SC}}_{n}$ in BS *f*, SIC is carried out at BS *f*. Based on the uplink NOMA protocol [35], the signal of MU with the highest channel gain will be first decoded at BS *f* and experiences interference from other MUs having relatively weaker channel gains on ${\mathrm{SC}}_{n}$. Therefore, the channel gains of MUs over ${\mathrm{SC}}_{n}$ in BS *f* are sorted as h1fn2h1fn2σfn2σfn2>h2fn2h2fn2σfn2σfn2>⋯hkfn2hkfn2σfn2σfn2>⋯hSfnfn2hSfnfn2σfn2σfn2, where $S_{fn}=\left|S_{fn}\right|$. Then the transmit data rate of ${\mathrm{MU}}_{k}$ associated with BS *f* over ${\mathrm{SC}}_{n}$ can be expressed as

(3) $$R_{kfn}=B\log_{2}\left(1+\frac{p_{kfn}H_{kfn}}{1+I_{kfn}+\phi_{kfn}}\right),$$

where Hkfn=hkfn2hkfn2σfn2σfn2. $I_{kfn}$ is the interference that ${\mathrm{MU}}_{k}$ receives from other MUs whose channel gains are smaller than that on ${\mathrm{SC}}_{n}$ of BS *f*, which can be given by

(4) $$I_{kfn}=\sum_{i\in \left\{S_{fn}\left|H_{ifn}\right.<H_{kfn}\right\}}p_{ifn}H_{ifn}.$$

$\phi_{kfn}=\sum_{f^{\prime }\in \left\{F\backslash f\right\}}\sum_{i\in S_{f^{\prime }n}}p_{if^{\prime }n}H_{ifn}$ is the interference from MUs associated to other BSs on ${\mathrm{SC}}_{n}$. Then the data rate of MUs at ${\mathrm{SC}}_{n}$ is

(5) $$R_{n}=B\sum_{f\in F}\sum_{k\in S_{fn}}\log_{2}\left(1+\frac{p_{kfn}H_{kfn}}{1+I_{kfn}+\phi_{kfn}}\right).$$

### 2.3. Problem Formation

In this paper, we focus on the EE maximization problem for all MUs considering the minimum data rate requirements of them. The MU association contains two parts: BS selection and sub-channel allocation. For a given MU association, the out-of-cell interference only come from the MUs associated with different BSs at the same sub-channel due to the orthogonality among the sub-channels. Then, each MU may not concern the whole EE, but the sub-channel EE it chooses. Therefore, the optimization problem is converted into solving the EE maximization of each sub-channel by appropriate MU association including BS selection and sub-channel allocation and power control.

From a physical standpoint, the efficiency with which a system uses a given resource is the ratio between the benefit obtained by using the resource and the corresponding incurred cost [17]. Applying this general definition to the uplink communication at ${\mathrm{SC}}_{n}$, then EE of ${\mathrm{SC}}_{n}$ can be written as

(6) $$EE_{n}=\frac{R_{n}}{\sum_{f\in F}\sum_{k\in S_{fn}}p_{kfn}+P_{c}},$$

where $P_{c}$ is the additional circuit power consumption over each sub-channel. Then the considered EE optimization problem can be formulated as

(7a) $$\begin{array}{c} \max_{P}\sum_{n\in N}EE_{n} \end{array}$$

(7b) $$\begin{array}{c} s.t.\;R_{kfn}\geq \sum_{j\in U_{j_{k}}}R_{j_{k}}+R_{req},\forall k\in K,n\in N, \end{array}$$

(7c) $$\begin{array}{c} \;\;\;p_{kfn}>0,p_{kfn}\leq P_{max},\forall k\in K,f\in F,n\in N, \end{array}$$

where P is the transmit power vector with elements $p_{kfn}$; $P_{max}$ is the maximum transmit power of each MU; and $R_{req}$ is the minimum data rate requirement of a MU. Since each MU is regarded as an MTCG for MTCDs, they should ensure the data rate that MTCDs can be uploaded to the SBS, therefore we have constraint (7b) as the data rate requirement of ${\mathrm{MU}}_{k}$ associated to BS *f* at ${\mathrm{SC}}_{n}$ [22]. Constraint (7c) is used to guarantee the feasible value ranges of P.

## 3. MU Association

The MTCDs associated to the corresponding MU are known beforehand. Since solving the optimization problem is equal to obtain the optimal EE of each sub-channel, the MU association will become the matching problem among BSs, sub-channels and MUs to achieve sub-channel EE maximization. Thus, we propose a MU association algorithm using matching game [30] in the following parts.

### 3.1. Matching Problem Formulation

To develop a low-complexity MU association algorithm, we first regard a sub-channel and a BS as a couple, denoted as (n,f). Then, the optimization problem is transformed to match the MUs to the couples and allocate power appropriately, such that the EE can be maximized. Finally, the matching problem is a many-to-one problem between MUs and couples based on matching game, which is described as follows.

**Definition** **1.** *Given two disjoint sets,*$K=\left\{1,\cdots ,K\right\}$*denotes the set for MUs, and*$M=\left\{\left(1,1\right)\right.\left(1,2\right)\cdots ,\left(2,1\right)\left.\cdots ,(n,f),\cdots (N,F)\right\}$*represents the couples. A many-to-one matching *Ψ* is a mapping from the set*K∪M*into the set of all subsets of*K∪M*for*f∈F, k∈K, n∈N*satisfying*

*i)*  Ψ(k)∈M;
*ii)*  Ψ(n,f)⊂K;
*iii)*  $\left|\Psi (k)\right|=1\left|,\Psi (SC_{n},f)\right|=S_{fn}$;
*iv)*  (n,f)=Ψ(k)⇔k∈Ψ(n,f).

Condition i indicates that each MU matches with a sub-channel-BS couple. On the other hand, each couple matches a subset of MUs, which is illustrated in condition ii. Condition iii states a MU can only associate one BS and choose one sub-channel while each couple matches $S_{fn}$ MUs.

The aim of each couple is to maximize its own EE. To this end, we exploit the swap operation into our matching algorithm. A swap operation means two MUs matching with different couples exchange their matchings based on different cases, while other MUs remain their matchings. The EE of the exchanged couples will be recomputed by the power control algorithm. Note that how to allocate power to obtain the optimal EE for a given sub-channel will be presented in the next section, and we assume it is known in advance. A swap operation will be approved and the matching will be exchanged only when all EE of the sub-channels belonging to the exchanged couples increase if the swap is performed. The swap operation will be continued until no swap is further preferred. More details are described in Algorithm 1.

 **Algorithm 1** The MU association matching algorithm.** Initialization phase:**$L=\left\lceil \frac{K}{NF}\right\rceil$, $\hat{K}=K$1: **for**
l=1:L
**do**2:  $\hat{M}=M$, Count=1
3:  **while** (Count≤M) **do**4:   $H_{k^{*}f^{*}n^{*}}=argmax{\left\{H_{kfn}\right\}}_{\forall k\in \hat{K},\left(f,n\right)\in \hat{M}}$. Assign $k^{*}$ to the couple $\left(f^{*},n^{*}\right)$, $\hat{K}=\hat{K}\backslash k^{*}$, $\hat{M}=\hat{M}\backslash \left(f^{*},n^{*}\right)$, and set Count=Count+15:  **end while**6: **end for**  **Swap matching phase:**
Indicator=17: **while** (Indicator) **do**8:  **for**
u=1:K
**do**9:   **for**
k=1:K
**do**10:    **if**
Ψ(k)=Ψ(u)
**then**11:     continue;12:    **else if**
${\mathrm{MU}}_{k}$ and ${\mathrm{MU}}_{u}$ are both in the coverage of the BSs of each other **then**13:     **switch** (Ψ(k),Ψ(u))14:     **case**
${\mathrm{MU}}_{k}$ and ${\mathrm{MU}}_{u}$ belonging to the same BS and different sub-channels: 15:      Calculate and compare the EE of the two sub-channels before and after the swap using the power control algorithm. If the EE of the two-subchannels both improve, exchange the sub-channel, form the new couple, and set Indicator=1. 16:     **case**
${\mathrm{MU}}_{k}$ and ${\mathrm{MU}}_{u}$ belonging to the different BSs and different sub-channels: 17:      Calculate and compare the EE of the two sub-channels before and after the swap using the power control algorithm. If the EE of the two sub-channels both improve, exchange the couple, form the new couple, and set Indicator=1. 18:     **case**
${\mathrm{MU}}_{k}$ and ${\mathrm{MU}}_{u}$ belonging to the different BSs and same sub-channels: 19:      Calculate the EE of the sub-channel before and after the swap using the power control algorithm. If the EE of the sub-channel has been improved, exchange the BS, form the new couple, and set Indicator=1.20:      **end switch**21:     **end if**22:    **end for**23:   **end for**24: **end while**

### 3.2. Matching Algorithm

Algorithm 1 contains a initialization phase and a swap matching phase. Considering the user fairness, the number of MUs accommodated by one sub-channel in a given BS is at most $\left\lceil \frac{K}{FN}\right\rceil$. In the initialization step, the basic idea is to associate the MU to the couple providing the largest channel gain. This will lead to either a higher data rate for the MU, or a lower transmit power. Since the value of sub-channel gain between MU and the uncovered BSs is invalid and the maximized sub-channel gain is always chosen, there is no need to know whether the MUs are in the coverage of the exchanged BSs. However, in the swap matching phase, this judgment should be considered at first to avoid the invalid swap. Then, exchange will happen in the three cases. Iterations will continue until no swap operation can be approved in a new round.

### 3.3. Convergence and Complexity

**Theorem** **1.** *The proposed MU association and power control algorithm converges after a finite number of swap operations.*

**Proof** **of Theorem 1.** For each swap operation, the matching changes from $\Psi_{ex}$ to $\Psi_{now}$. We have $EE_{n,ex}$ and $EE_{n,now}$ to denote the corresponding EE of of $\Psi_{ex}$ and $\Psi_{now}$ on ${\mathrm{SC}}_{n}$. Based on the aim of swap operation, we have $EE_{n,now}>EE_{n,ex}$, that is, the EE of each sub-channel increases after each swap matching. Since each sub-channel is orthogonal to each other, the system EE will increase owing to the improved EE of each sub-channels. Moreover, the system EE has an upper bound due to the limited transmit power of each MU. Therefore, the MU association algorithm and power allocation converge after a finite number of swaps. □

## 4. Power Control

In this section, we will investigate the optimal power control design appearing in Algorithm 1 to obtain the maximum EE of $SC_{n}$. Before we present the optimization problem for $EE_{n}$ maximization, we first deal with $R_{n}$, which can be rewritten as

(8) $$R_{n}\left(p_{fn}\right)=B\sum_{f\in F}\log_{2}\left(1+\frac{\sum_{k\in S_{fn}}p_{kfn}H_{kfn}}{1+\phi_{kfn}}\right),$$

and we can also obtain

(9) $$R_{tot,k}=\sum_{j\in U_{k}}R_{j_{k}}=\log_{2}\left(1+\sum_{j\in U_{k}}H_{j_{k}k}q_{j_{k}}\right).$$

Due to the multi-interference in the sum-rate function in (8), $EE_{n}$ in (6) is non-convex and cannot be directly solved by the generalized fractional programming approach. Then, we first transform the numerator into the difference of two non-negative functions and the $EE_{n}$ maximization can be rewritten as

(10a) $$\begin{array}{cc} \; & \max_{p_{fn}}\;{\tilde{\eta }}_{n}=\frac{F^{+}\left(p_{fn}\right)-F^{-}\left(p_{fn}\right)}{\sum_{f\in F}\sum_{k\in S_{fn}}p_{kfn}+P_{c}} \end{array}$$

(10b) $$\begin{array}{cc} \; & s.t.\;(7c),(11), \end{array}$$

with

(11) $$C_{{}_{kfn}}^{+}\left(p_{fn}\right)-C_{{}_{kfn}}^{-}\left(p_{fn}\right)\geq 0,\;\forall k\in K,\forall f\in F,\forall n\in N,$$

where $p_{fn}=[p_{1fn},p_{2fn},\cdots ,{p_{k}}_{fn},\cdots ,{p_{S_{fn}}}_{Fn}{]}^{T}$ denotes the transmit power vector for MUs on $SC_{n}$. Moreover, we have

(12) $$\begin{array}{c} F^{+}\left(p_{fn}\right)=B\sum_{f\in F}\log_{2}\left(1+\sum_{k\in S_{fn}}p_{kfn}H_{kfn}+\phi_{kfn}\right),\\ F^{-}\left(p_{fn}\right)=B\sum_{f\in F}\log_{2}\left(1+\phi_{kfn}\right),\\ C_{{}_{kfn}}^{+}\left(p_{fn}\right)=B\log_{2}\left(1+p_{kfn}H_{kfn}\right.+I_{kfn}+\left.\phi_{kfn}\right)\\ \;\;\;\;\;\;-R_{tot,k}-R_{req},\\ C_{{}_{kfn}}^{-}\left(p_{fn}\right)=B\log_{2}\left(1+I_{kfn}+\phi_{kfn}\right). \end{array}$$

Note that $F^{+},F^{-},C^{+},\mathrm{and}\;C^{-}$ are concave functions regarding to $p_{fn}$, then the numerator of (10a) and the constraint functions in (10b) are expressed as the difference of concave functions, which are not concave in general. Motivated by [31,32], where sequential optimization is used to solve the similar problem as (10), we adopt this method and combine it with fractional programming and exhaustive search to propose two power control algorithms. Before introducing the two algorithms, we first present the details of the sequential optimization theory in the next sub-section.

### 4.1. Sequential Optimization Theory

Sequential optimization is a powerful tool that can tackle a difficult optimization problem by solving a sequence of approximate problems in simple forms with affordable complexity. Specifically, we give a formal maximization problem $\bar{F}$ with a compact feasible set as [31,32], shown as

(13a) $$\begin{array}{c} \max_{x}f_{0}\left(x\right) \end{array}$$

(13b) $$\begin{array}{c} s.t.\;f_{i}\left(x\right)\geq 0,\forall i\in \left\{1,\cdots ,I\right\}, \end{array}$$

where $f_{0}\left(x\right)$ is the differentiable objective with constraints $f_{i}\left(x\right)\geq 0$. Let $G^{\left(v\right)}$ be the problem solved in the *v*-th iteration by the sequential method to tackle problem $\bar{F}$, which can be written as

(14a) $$\begin{array}{c} \max_{x}g_{0}^{\left(v\right)}\left(x\right) \end{array}$$

(14b) $$\begin{array}{c} s.t.\;g_{i}^{\left(v\right)}\left(x\right)\geq 0,\forall i\in \left\{1,\cdots ,I\right\}, \end{array}$$

where $g_{0}^{\left(v\right)}\left(x\right)$ is the differentiable objective with the constraints $g_{i}^{\left(v\right)}\left(x\right)$. Then, if $g_{0}^{\left(v\right)}\left(x\right)$ and $g_{i}^{\left(v\right)}\left(x\right)$ are suitable continuous functions and constraints, they must satisfy the following two properties:

1) $g_{0}^{\left(v\right)}\left(x\right)\leq f_{0}\left(x\right)$, $g_{i}^{\left(v\right)}\left(x\right)\leq f_{i}\left(x\right)\;\forall x$;
2) $g_{0}^{\left(v\right)}\left({\left(x^{*}\right)}^{\left(v-1\right)}\right)=f_{0}\left({\left(x^{*}\right)}^{\left(v-1\right)}\right)$, $g_{i}^{\left(v\right)}\left({\left(x^{*}\right)}^{\left(v-1\right)}\right)$
  $\leq f_{i}\left({\left(x^{*}\right)}^{\left(v-1\right)}\right)$.

${\left(x^{*}\right)}^{\left(v-1\right)}$ is the optimal solution of the problem solved at iteration (v−1)-th. This means the solution sequence ${\left\{\left(x^{*}\right)\right\}}^{\left(v\right)}$ of (14) monotonically increases the value of (13), i.e., $f_{0}\left({\left(x^{*}\right)}^{\left(v\right)}\right)\geq f_{0}\left({\left(x^{*}\right)}^{\left(v-1\right)}\right)$ for all *v*, which guarantees the convergence of the sequential method. Next, if the following third property is also satisfied:

3) $\nabla g_{0}^{\left(v\right)}\left({\left(x^{*}\right)}^{\left(v-1\right)}\right)=f_{0}\left({\left(x^{*}\right)}^{\left(v-1\right)}\right)$, $\nabla g_{i}^{\left(v\right)}\left({\left(x^{*}\right)}^{\left(v-1\right)}\right)=\nabla f_{i}\left({\left(x^{*}\right)}^{\left(v-1\right)}\right)$.

then every limit point of ${\left\{x\right\}}^{\left(v\right)}$ of (14) fulfills the Karush–Kuhn–Tucker (KKT) conditions of problem $\bar{F}$ in (13).

Therefore, if a maximization problem finds suitable approximate problems which can fulfill the above three properties, its optimal value can be approximated by solving the monotonically increased sequential problems. The critical issue is that the suitable approximate problems are solved easier than the original problem. In the rest of this section, we will first find the sequential approximate problems to the numerator in problem (10).

### 4.2. Sequential Fractional Power Control Algorithm

Based on sequential optimization, we should find a sequence problem to approximate optimization problem (10). To circumvent this issue, we obtain the following main result with the first-order Taylor expansion at ${p_{fn}}^{\left(v\right)}$ of $F^{-}\left(p_{fn}\right)$.

**Proposition** **1.** *For any given*${p_{fn}}^{\left(v\right)}$, *the sequence approximation problem of (10), denoted by*$G^{\left(v\right)}$*can be written as*

(15a) $$\begin{array}{cc} \; & \max\;\eta_{n}=\frac{F^{+}\left(p_{fn}\right)-\tilde{F}\left(p_{fn}\right)}{\sum_{f\in F}\sum_{k\in S_{fn}}p_{kfn}+P_{c}} \end{array}$$

(15b) $$\begin{array}{cc} \; & s.t.\;C_{{}_{kfn}}^{+}\left(p_{fn}\right)-\tilde{C}\left(p_{fn}\right)\geq 0, \end{array}$$

(15c) $$\begin{array}{cc} \; & \;\;\left(7c\right), \end{array}$$

*with optimal solution*${p_{fn}^{*}}^{\left(v\right)}$, *where*

(16) $$\begin{array}{cc} \tilde{F}\left(p_{fn}\right) & =F^{-}\left({p_{fn}}^{\left(v\right)}\right)\\ \; & -{\left(\nabla F^{-}\left({p_{fn}}^{\left(v\right)}\right)\right)}^{T}\left(p_{fn}-{p_{fn}}^{\left(v\right)}\right) \end{array}$$

(17) $$\begin{array}{cc} \tilde{C}\left(p_{fn}\right) & =C_{{}_{kfn}}^{-}\left({p_{fn}}^{\left(v\right)}\right)\\ \; & -{\left(\nabla C^{-}\left({p_{fn}}^{\left(v\right)}\right)\right)}^{T}\left(p_{fn}-{p_{fn}}^{\left(v\right)}\right) \end{array}$$

*If*$p_{fn}^{\left(v\right)}={p_{fn}^{*}}^{\left(v-1\right)},\forall v\geq 1$, *then*${\left\{\eta_{n}{p_{fn}^{*}}^{\left(v\right)}\right\}}^{\left(v\right)}$*is monotonically increasing and converges to a value*${\tilde{\eta }}_{n}$. *Furthermore, any limit point of sequence*${\left\{\eta_{n}{p_{fn}^{*}}^{\left(v\right)}\right\}}^{\left(v\right)}$*that achieves*${\tilde{\eta }}_{n}$*fulfills the KKT optimality conditions of (10a).*

**Proof** **of Proposition 1.** As we know, any concave function is the upper-bounded of its first-order Taylor expansion at any point. Since $F^{-}\left(p_{fn}\right)$ and $C^{-}\left(p_{fn}\right)$ are concave functions, for any power vector $p_{fn}^{\left(v\right)}$ we have

(18) $$\begin{array}{cc} \; & F^{+}\left(p_{fn}\right)-F^{-}\left(p_{fn}\right)\\ \; & \geq F^{+}\left(p_{fn}\right)-\tilde{F}\left(p_{fn}\right)\\ \; & =F^{+}\left(p_{fn}\right)-F^{-}\left({p_{fn}}^{\left(v\right)}\right)\\ \; & -{\left(\nabla F^{-}\left({p_{fn}}^{\left(v\right)}\right)\right)}^{T}\left(p_{fn}-{p_{fn}}^{\left(v\right)}\right), \end{array}$$

(19) $$\begin{array}{cc} \; & C^{+}\left(p_{fn}\right)-C^{-}\left(p_{fn}\right)\\ \; & \geq C^{+}\left(p_{fn}\right)-\tilde{C}\left(p_{fn}\right)\\ \; & =C^{+}\left(p_{fn}\right)-C_{{}_{kfn}}^{-}\left({p_{fn}}^{\left(v\right)}\right)\\ \; & -{\left(\nabla C^{-}\left({p_{fn}}^{\left(v\right)}\right)\right)}^{T}\left(p_{fn}-{p_{fn}}^{\left(v\right)}\right). \end{array}$$

Hence, (15a) and (15b) are lower bounds of (10a) and (11), respectively. Since the lower bounds in (16) are tight when evaluated by $p_{fn}^{\left(v\right)}$, it follows that (15a) and (15b) are equal to (10a) and (11), respectively, for $p_{fn}=p_{fn}^{\left(v\right)}$. Similarly, the gradients of (15a) and (15b) are equal to those of (10a) and (11), for $p_{fn}=p_{fn}^{\left(v\right)}$. Thus, (15) fulfills all the properties described in the above sub-section, which completes the proof of this proposition. □

For any $p_{fn}^{\left(v\right)}$, problem (15) has a concave numerator and an affine denominator, while the constraint functions in (15b) and (15c) are both concave and affine. Therefore, (15) is a single-ratio problem, which can be solved by the generalized fractional programming. We adopt the widely used Dinkelbach’s algorithm to solve it. According to Dinkelbach’s method [33], we first introduce the following auxiliary function

(20) $$T\left(p_{fn},\eta_{n}\right)=f_{n}\left(p_{fn}\right)-\eta_{n}g_{n}\left(p_{fn}\right),$$

with $f_{n}\left(p_{fn}\right)=F^{+}\left(p_{fn}\right)-\tilde{F}\left(p_{fn}\right)$, and $g_{n}\left(p_{fn}\right)=\sum_{f\in F}\sum_{k\in S_{fn}}p_{kfn}+P_{c}$.

**Theorem** **2.** *Let*$\eta_{n}^{*}$, $p_{fn}^{*}$and$p_{kfn}^{*}$denote the optimal value, optimal solution and its elements of problem (15), respectively. Then, we have

(21) $$\begin{array}{c} \eta_{n}^{*}=\frac{F^{+}\left(p_{fn}^{*}\right)-\tilde{F}\left(p_{fn}^{*}\right)}{\sum_{f\in F}\sum_{k\in S_{fn}}p_{kfn}^{*}+P_{c}}=\max\;\frac{F^{+}\left(p_{fn}\right)-\tilde{F}\left(p_{fn}\right)}{\sum_{f\in F}\sum_{k\in S_{fn}}p_{kfn}+P_{c}}, \end{array}$$

*if and only if*

(22) $$\begin{array}{c} \max\left\{\right.T\left(p_{fn},\eta_{n}^{*}\right)=f_{n}\left(p_{fn}\right)-\eta_{n}^{*}g_{n}\left(p_{fn}\right)\left.\right\}\\ \;\;\;\;\;\;\;\;=f_{n}\left(p_{fn}^{*}\right)-\eta_{n}^{*}g_{n}\left(p_{fn}^{*}\right)=0. \end{array}$$

**Proof** **of Theorem 2.** Theorem 2 was proved in [33,36], and we omit it due to the limited space. □

The optimal $\eta_{n}^{*}$ can be obtained by Dinkelbach’s method, which is summarized in Algorithm 2. As shown in the algorithm, we need to solve the problem (23) for a given parameter ${\eta_{n}}^{\left(c\right)}$ in each iteration. In Algorithm 2, $\eta_{n}$ has been updated as ${\eta_{n}}^{\left(c\right)}$ in each iteration until convergence. or reaching the maximum number of iterations. ${p_{fn}}^{\left(c\right)}$ denotes the optimal power of the following problem in the *c*-th iteration, which can be obtained in Algorithm 3, as given by

(23) $$\begin{array}{c} \underset{p_{fn}}{\max}T\left(p_{fn},{\eta_{n}}^{\left(c\right)}\right)=f_{n}\left(p_{fn}\right)-{\eta_{n}}^{\left(c\right)}g_{n}\left(p_{fn}\right)\\ s.t.\;C_{{}_{kfn}}^{+}\left(p_{fn}\right)-\tilde{C}\left(p_{fn}\right)\geq 0,\\ \;\;\left(7c\right), \end{array}$$

 **Algorithm 2** The Dinkelbach’s algorithm. **Initialization phase:** Set iteration c=1, ${\eta_{n}}^{\left(c\right)}>0$, the maximum number of iterations $C_{\max}$, and error tolerance τ>0.1: **repeat**
2: Solve the equivalent problem (23) for a given ${\eta_{n}}^{\left(c\right)}$ to obtain the solution ${p_{fn}}^{\left(c\right)}$.3: ${\eta_{n}}^{\left(c\right)}=\frac{F^{+}\left({p_{fn}}^{\left(c\right)}\right)-\tilde{F}\left({p_{fn}}^{\left(c\right)}\right)}{\sum_{f\in F}\sum_{k\in S_{fn}}p_{kfn}{}^{\left(c\right)}+P_{c}}$,4: c=c+1.5: **until**
$\left|T({\eta_{n}}^{\left(c-1\right)},{p_{fn}}^{\left(c-1\right)})\right|\leq \tau$
**or**
$c>C_{\max}$
6: $\eta_{n}^{*}={\eta_{n}}^{\left(c-1\right)}$, $p_{fn}^{*}={p_{fn}}^{\left(c-1\right)}$.

 **Algorithm 3** The algorithm for solving problem (23). **Initialization phase:** Set $p_{fn}^{\left(0\right)}$, iteration index v=0, the maximum iterations $V_{\max}$ and error tolerance μ. Calculate $f_{n}\left({p_{fn}}^{\left(0\right)}\right)\;\;-\;\;{\eta_{n}}^{\left(c\right)}g_{n}\left({p_{fn}}^{\left(0\right)}\right)$.1: **repeat**
2: Solve the problem (23) to obtain the optimal solution $p_{fn}^{*}$ for given $p_{fn}^{\left(v\right)}$ and ${\eta_{n}}^{\left(c\right)}$.3: v=v+1.4: Set $p_{fn}^{\left(v\right)}\;\;=\;\;\;p_{fn}^{*}$ and cacluate $f_{n}\left({p_{fn}}^{\left(v\right)}\right)\;\;-\;{\eta_{n}}^{\left(c\right)}g_{n}\left({p_{fn}}^{\left(v\right)}\right)$.5: **until**
$\left|f_{n}\left({p_{fn}}^{\left(v\right)}\right)-{\eta_{n}}^{\left(c\right)}g_{n}\left({p_{fn}}^{\left(v\right)}\right)\right.-\left.\left(f_{n}\left({p_{fn}}^{\left(v-1\right)}\right)-{\eta_{n}}^{\left(c\right)}g_{n}\left({p_{fn}}^{\left(v-1\right)}\right)\right)\right|\leq \mu$
6: $p_{fn}^{*}{=p}_{fn}^{\left(v\right)}$.

### 4.3. Computational Complexity Analysis

In above sub-section, we have proposed the SFPCA including two steps, i.e., Algorithms 2 and 3. The computational complexity of them are separately discussed. First, we use *C* to denote the number of iterations for Algorithm 2, where *C* is bounded by $C_{max}$. From Section V, we can see that Algorithm 2 will converge after a few number of iterations. Then we discuss the computational complexity of Algorithm 3, the complexity of this algorithm is mainly caused by (23), and denoted by X. The computational complexity of (23) is $O({S_{n}}^{3})$ [37], where $S_{n}$ is the number of MUs at ${\mathrm{SC}}_{n}$. The complexity of Algorithm 3 is $X=VO({S_{n}}^{3})$, where *V* is the the number of iterations bounded by $V_{max}$. In summary, the computational complexity of the power control algorithm is O(CX).

### 4.4. Sequential Exhaustive Algorithm

To evaluate the performance of the SFPCA, a sequential exhaustive algorithm (SEA) combined with sequential optimization and exhaustive search is proposed in this section. The detailed procedures of the compared algorithm is illustrated as follows. To solve the problem in an easier manner, we introduce the auxiliary variable $y_{n}$, as given by

(24) $$\begin{array}{c} y_{n}=B\sum_{f\in F}\log_{2}\left(1+\frac{\sum_{k\in S_{fn}}p_{kfn}H_{kfn}}{1+\phi_{kfn}}\right). \end{array}$$

if we fix $y_{n}$, the objective function (15a) can be recast as

(25) $$\begin{array}{c} \max_{p_{fn}}\frac{y_{n}}{\sum_{f\in F}\sum_{k\in S_{fn}}p_{kfn}}\\ s.t.\sum_{f\in F}\log_{2}\left(1+\frac{\sum_{k\in S_{fn}}p_{kfn}H_{kfn}}{1+\phi_{kfn}}\right)\geq y_{n}. \end{array}$$

Due to the multi-user interference, we cannot solve problem (25) by standard convex optimization tools. Similar to SFPCA, sequential optimization is applied and the approximate problem can be shown as

(26a) $$\max_{p_{fn}}\frac{F^{+}\left(p_{fn}\right)-\tilde{F}\left(p_{fn}\right)}{\sum_{f\in F}\sum_{k\in S_{fn}}p_{kfn}+P_{c}}$$

(26b) $$s.t.\;F^{+}\left(p_{fn}\right)-\tilde{F}\left(p_{fn}\right)\geq y_{n},\forall n\in N,$$

(26c) (7c),(11).

It can be observed that since $y_{n}$ is fixed, (26) is equivalent to minimize the linear function $\sum_{f\in F}\sum_{k\in S_{fn}}p_{kfn}+P_{c}$ in the denominator, subject to convex constraints. Then, problem (26) can be solved by plain convex programming. To implement an efficient line search for $y_{n}$, the bound of $y_{n}$ is given by

(27) $$\begin{array}{ccc} {\overset{⌣}{\;y}}_{n} & = & FS_{fn}R_{req}\\ & \leq & \sum_{f\in F}\log_{2}\left(1+\frac{\sum_{k\in S_{fn}}p_{kfn}H_{kfn}}{1+\phi_{kfn}}\right)\\ & < & \sum_{f\in F}\log_{2}\left(1+\sum_{k\in S_{fn}}P_{\max}H_{kfn}\right)\\ & = & {\overset{⌢}{\;y}}_{n}. \end{array}$$

Then, the optimial $p_{fn}$ can be obtained by searching an appropriate value of $y_{n}$ with stepsize ε.

## 5. Numerical Results and Discussions

In this section, the effectiveness of our proposed MU association and power control algorithms in M2M-enabled HetNets with NOMA was demonstrated by Monte Carlo simulations. The HetNets included one MBS and two SBSs, and the radius of the cells for them were 200 m and 80 m, respectively. MUs were randomly and uniformly distributed. The values of the simulation parameters are summarized in Table 1.

**Figure 2:**
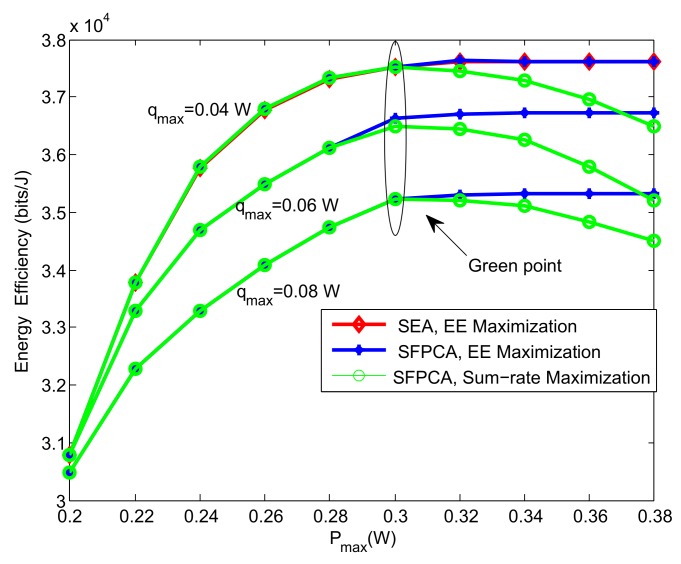
EE versus the maximum transmit power for different schemes.

We considered the EE performance obtained from EE maximization and sum-rate maximization with different $P_{max}$ in Figure 2. The latter could be obtained in the first iteration of Algorithm 2 due to $q^{\left(1\right)}=0$. In order to reflect the influence of $q_{max}$, we gave four schemes of different $q_{max}$. From the figure, we can see that all of the four schemes had a “green point”, where EE and sum rate could both achieve their optimal values. Different from sum rate, EE became gradually flat while sum rate decreased after “green point” as $P_{max}$ grew. The reason is when the maximum EE is achieved, no more transmit power is needed. For sum rate maximization, larger sum rate requires more transmit power, and its ratio (EE) may decrease, since the numerator (sum rate) and denominator (sum transmit power) both grow. We can also see that the EE decreased as $q_{max}$ increased, because the increase of $q_{max}$ means the data rate requirement of MUs increases. It is worth noting that even though MTCDs have lower data rate and transmit power, they can also have a strong influence on the overall uplink EE with their massive number. Furthermore, Algorithm 1 with higher EE had similar tendency as Algorithm 4, which proves the correctness of our algorithms.

**Figure 3:**
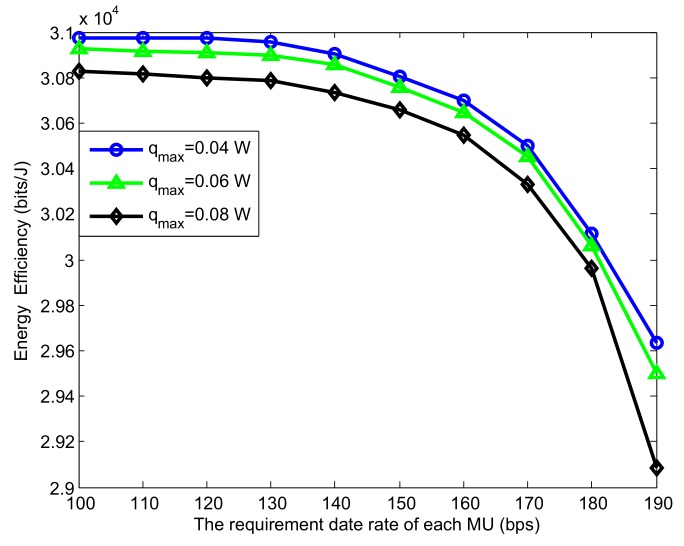
EE versus the date rate requirement of each MU for different schemes.

Figure 3 shows the EE performance with respect to different data rate requirements of MUs with the different transmit power of MTCDs. The four curves all decreased as $R_{req}$ increased. This is due to the fact that higher data rate will narrow the feasible value regions of the transmit power. Note that the four curves decreased slightly first, when $R_{req}=150\;\mathrm{bps}$, the EE of the four schemes all declined distinctly, since higher data rate requirement may require more transmit power, destroying the balance of sum transmit power and sum rate. As explained above, the increase of $q_{max}$ leads to the increase of data rate requirement, and the variation of EE is in line with the reason as the figure shown.

 **Algorithm 4** Sequential exhaustive algorithm.
** Initialization phase:**
 ε>0, $\omega =\frac{{\overset{⌢}{y}}_{n}-{\overset{⌣}{y}}_{n}}{\varepsilon }$1: **for**
$y_{fn}\in \left[{\overset{⌣}{y}}_{fn}:\omega :{\overset{⌢}{y}}_{fn}\right)$
**do**2:
$p_{fn}^{*}=\mathrm{argmin}\sum_{f\in F}\sum_{k\in S_{fn}}p_{kfn}+P_{c}$3: **end for**4: Obtain the optimal solution of $p_{fn}$.

**Figure 4:**
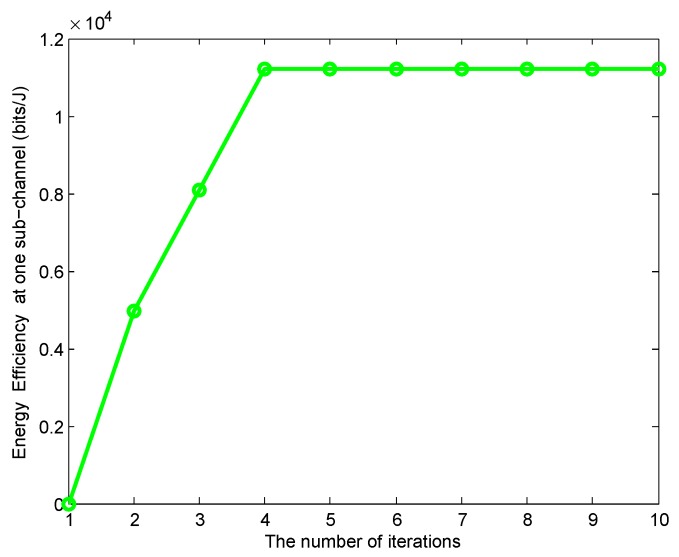
Convergence property of Algorithm 2.

**Figure 5:**
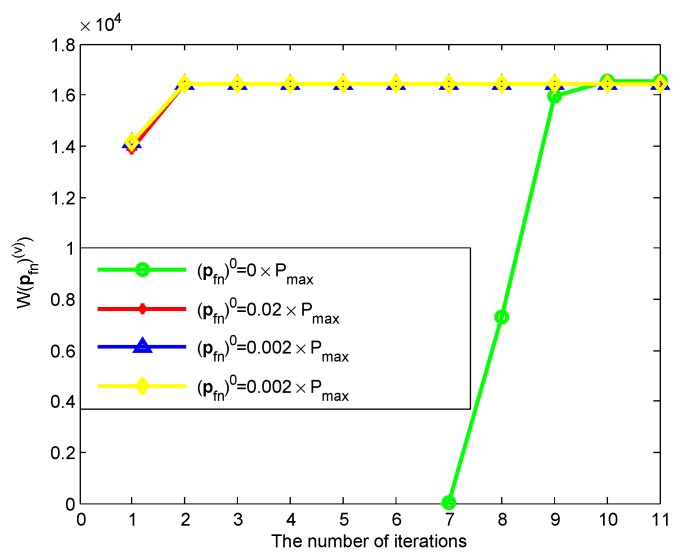
Convergence property of Algorithm 3.

Figure 4 and Figure 5 shows the convergence property of Algorithms 2 and 3. For simplicity, the numerical results in two figures are from a random chosen sub-channel, where ${\eta_{n}}^{\left(c\right)}=1$. In Figure 4, we can see that the number of iterations are limited within four times. To show the influence of $p_{fn}^{\left(0\right)}$, we give the different values of $p_{fn}^{\left(0\right)}$ in Figure 5, where $\left|f_{n}\left({p_{fn}}^{\left(v\right)}\right)-{\eta_{n}}^{\left(c\right)}g_{n}\left({p_{fn}}^{\left(v\right)}\right)\right.-\left.\left(f_{n}\left({p_{fn}}^{\left(v-1\right)}\right)-{\eta_{n}}^{\left(c\right)}g_{n}\left({p_{fn}}^{\left(v-1\right)}\right)\right)\right|=W({p_{fn}}^{\left(v\right)})$. It is shown that the initial values have an effect on the number of iterations. Specifically, when $p_{fn}{}^{\left(0\right)}=0\times P_{\max}$, less than 11 times is needed to reach the convergence. Although the initial values affect the number of iterations, it does not affect the final results.

**Figure 6:**
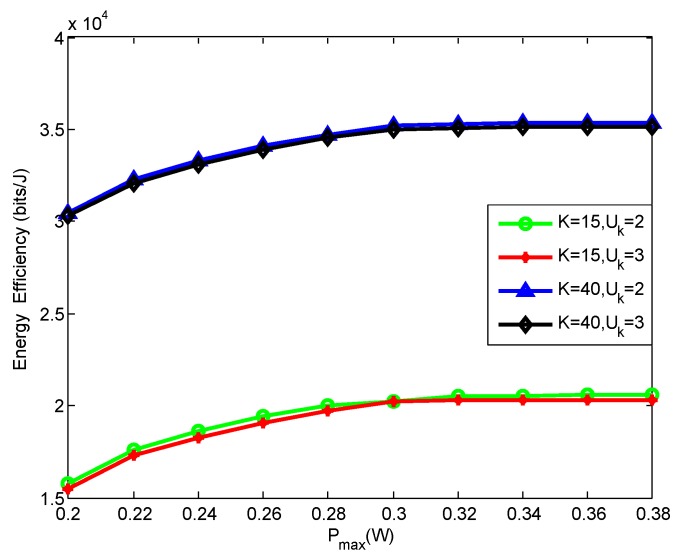
EE versus the maximum transmit power for the different number of MUs and MTCDs.

To show the relationship between the different numbers of MUs and MTCDs and the EE, we have Figure 6. It is not surprise to see that the EE performance of all these schemes increases as $P_{max}$ grows. From the four schemes, we can find out that the EE of K=40 is much larger than that of K=15, since the NOMA scheme can obtain much higher EE by supporting multiple MUs, and they can choose the suitable couples by swap operations for better EE. From the Algorithm 1, we know that power control algorithm needs to be executed after each swap operation, that is, the number of iterations increases with the increase of *K* and more process time are required. From the Figure 6, we can also see that the EE of $U_{k}=2$ is larger than that of $U_{k}=3$ under the same *K*, i.e., *K* has much greater impact on EE than $U_{k}$, since the NOMA scheme can obtain much higher EE by supporting multiple MUs and the increasing $U_{k}$ represents the increase data rate requirement of MUs.

**Figure 7:**
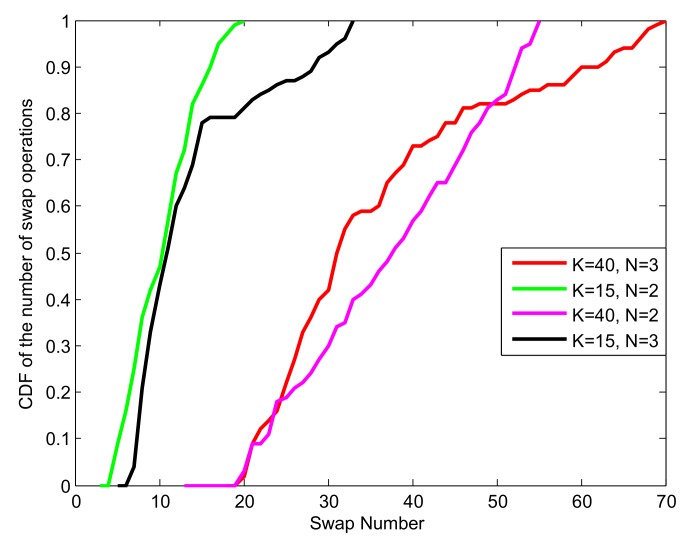
CDF of the number of swap operations for convergence.

Figure 7 presents the cumulative distribution function (CDF) of the number of swap operations of different scenarios when the matching algorithms reached convergence. From the figure we can see that more swap operations were needed for a larger number of MUs and sub-channels, such as, K=40,N=3 needed more swap operations than that K=40,N=2 and K=15,N=3. Especially, less than 70 swap operations were needed for K=40 and N=3.

## 6. Conclusions

This work investigated the uplink EE maximization problem in M2M-enabled HetNets with NOMA, where a MU acting as an MTCG can decode and forward both the information of MTCDs and its own data to the BS directly. Due to the limited spectrum resource, each BS shared the same sub-channels and NOMA was adopted between MUs in the same BS and sub-channel. The EE maximization problem was formulated, where MU association and power control were combined with each other. To solve it, a MU association matching algorithm was proposed based on the matching game. Under a given MU association, the uplink EE maximization was transformed into the EE maximization of each sub-channel. Two power control algorithms were provided to obtain the suboptimal power solutions based on sequential optimization. Simulation results showed that our proposed algorithms performed better than EE performance. It is known that cellular network is a key way to connect the M2M communications to the core network; our proposed scheme provided a new strategy for MTCDs to connect the cellular network with regard to MUs as their MTCGs based NOMA, and the power control of MTCDs was also considered as the constraints for the EE optimization problem. In fact, large scale devices are a more realistic scenario for 5G and next generation network, and since the number of MTCDs is considered on a small scale in this paper, the extension of our algorithms for large scale devices is one of the future works. Furthermore, the research of high computation complexity of the proposed algorithms for large scale devices is also a significant problem.

## Figures and Tables

**Table 1:** Simulation parameters.

Parameters	Meanings	Values
*F*	Number of BSs	3
*B*	The frequency bandwidth of each sub-channel	15kHz
*K*	Number of MUs	40
$U_{k}$	Number of MTCDs of each MU	2
$\sigma_{fn}^{2}$	Noise variance	2dBm
μ,τ	Error tolerance	$10^{-3}$
$P_{max}$	The maximum of transmit power of MU	0.2W
$q_{max}$	The maximum of transmit power of MTCD	0.08W
α	Path loss factor	3
Pc	The circuit power at each sub-channel	0.1W
$R_{req}$	The data rate requirement of each MU	100bps
